# Reverse Osmosis Coupled with Ozonation for Clean Water Recovery from an Industrial Effluent: Technical and Economic Analyses

**DOI:** 10.3390/membranes15010033

**Published:** 2025-01-16

**Authors:** Ivette Montero-Guadarrama, Claudia Muro Urista, Gabriela Roa-Morales, Edith Erialia Gutiérrez Segura, Vianney Díaz-Blancas, Germán Eduardo Dévora-Isiordia, Jesús Álvarez-Sánchez

**Affiliations:** 1 Tecnológico Nacional de México/Instituto Tecnológico de Toluca, Av. Tecnológico S/N, Col. Agrícola Bellavista, Metepec C.P. 52149, Estado de México, Mexico; dd22281236@toluca.tecnm.mx (I.M.-G.); vianney.db@toluca.tecnm.mx (V.D.-B.); 2 Centro Conjunto de Investigación en Química Sustentable CCIQS UAEM-UNAM, Facultad de Química, Universidad Autónoma del Estado de México (UAEMex), Carretera Toluca-Atlacomulco, Km 14.5, Toluca C.P. 50200, Estado de México, Mexico; eegutierrezs@uaemex.mx; 3 Departamento de Ciencias del Agua y Medio Ambiente, Instituto Tecnológico de Sonora, 5 de Febrero 818 sur Col. Centro, Cd. Obregón C.P. 85000, Sonora, Mexico; german.devora@itson.edu.mx (G.E.D.-I.); jesus.alvarez@itson.edu.mx (J.Á.-S.)

**Keywords:** ozonation, activated carbon catalytic ozonation, reverse osmosis, technical feasibility, economic feasibility

## Abstract

Technical and economic criteria were used to evaluate the feasibility of the treatment of an industrial effluent (10 m^3^/h) for water recovery and reuse. The treatment evaluation included the following: (1) effluent characteristic determination; (2) selection and evaluation of the effluent treatment at lab scale, establishing operating conditions and process efficiency; (3) scaling up the treatment process to the industrial level; (4) treatment plant design and commercial availability analysis of the required equipment; and (5) the costs of the inversion and operation of the plant treatment, cost/m^3^ for water recovery, and time of investment recovery. The physicochemical characteristics of the effluent exposed the polluted wastewater with sodium chloride salts and colourants, predominating a mixture of tartrazine, Red 40, and brilliant blue from the synthesis of food additives. Other contributions of organic compounds and salts could be in minor content. According to the effluent conditions, a coupled process, integrated with ozonation and reverse osmosis, was indicated to be a treatment for water recovery. Scaling up the plant treatment design resulted in 130 m^2^ of area, producing 7.7 m^3^/h of clean water. The cost of the effluent treatment was 1.4 USD/m^3^, with an inversion return of 3.4 years and cost investment of USD 860,407. The treatment process resulted a viable project for water recovery.

## 1. Introduction

Industrial effluents are considered a source of water production because they can be treated for water recovery, which covers the necessities of water reuse in industry or water scarcity problems. However, the effluents and treatment process should be analyzed beforehand to comply with water recovery and reuse requirements.

Frequently, the effluent treatment process for water recuperation comprises coupled membrane processes or hybrid methods, including previous or posterior steps to membranes, to achieve high efficiency in the treatment. Previous steps alleviate the membrane operation and enable the membrane flux and the membrane fouling; posterior steps, complement the process, produce clean water with different reuse possibilities and efficiencies [1,2,3,4].

The selection of the treatment process for water recovery also requires studies of technical and economic viability for its implementation at the industrial level [5]; however, reports including this information are scarce. Therefore, more information is needed to know the feasibility of water recovery from industrial effluent treatment.

Current studies focused on the evaluation of coupled processes for water recovery are found in Venzke et al. [6]. They evaluated the treatment of a petrochemical effluent by reverse osmosis–electrodialysis reversal (RO-EDR). The process achieved 87.3% of the efficiency; the clean water was reused in cooling towers. Hernández et al. [1] treated a food effluent for water recovery using UF-RO. In this case, 60% of clean water was recovered with reuse possibilities in the industry. Santos et al. [7] investigated the membrane distillation MD coupled with filtration (F) (F-MD) for water recovery by the treatment of a petrochemical effluent. The recovered water was disposed of for direct use in the production process.

Ozbey-Unal et al. [3] analyzed effluent treatment from a PTAR using coagulation-MF-RO. The authors obtained 80% of RO efficiency. Günes and Gönder [8] evaluated the hybrid system’s efficiency, electrocoagulation–NF–RO (EC–NF–RO), from treated textile effluent for water recovery. The contaminants removal showed 93% COD, 99% conductivity, 97% chloride, and 91% TDS [7].

Mohan and Oke [9] investigated the treatment of a textile effluent using the Sedimentation Ozone–Sedimentation Multi-grade Filter–Three stage of RO (S-Oz-SF-RO). The authors recovered 157 m^3^/day of clean water from 750 m^3^/day of effluent fed. The water reuse was established as cooling water and boiler water. Hernández et al. [4] treated a food effluent, achieving 80% of clean water production, using a Sedimentation-Activated carbon filter–Ion exchange resins–RO (S-ACF-IEX-RO).

In turn, cost information on water recovery processes is available from Li et al. [10]. The authors evaluated the cost of a coupled system of the bioreactors MBR and NF (MBR-NF) to treat an effluent, resulting in 1.24 MUS/year and a payback period of 3.11 years.

Nazia et al. [11] treated old Industrial Landfill Leachate using coagulation–UF (C-UF). As a result, 70% of water was recovered for different industrial applications. The cost of water production was estimated to be 1.45 USD/m^3^ with a payback period of 1.38 years. Ankoliya et al. [12] treated an effluent from the dairy industry using the coupled process BRO-BMED. The cost of water recovery resulted in 0.77 USD/L.

Oke et al. [13] estimated the cost of treatment using a brewery industry effluent. The investment in annual production for 45,000,000 L/year was USD 2,416,358, with a payback period of 3.27 years. Tripathi et al. [14] analyzed the effluent treatment from the textile industry, comparing the use of single ozonation (SO) and coupled SO–bioreactors (SO-BR). The cost analysis resulted in SO 29.69 USD/year, whereas the coupled system was the most efficient and economically viable (19 USD/year, saving 34%). Montero-Guadarrama et al. [5] evaluated the treatment of a food industrial effluent for clean water recovery. The investment cost was USD 565,569.00 for treat 250 m^3^/day, with a payback period of 2.3 years or 1.5 USD/m^3^.

Currently, Barros et al. [15] studied a coupled process of ultrafiltration, reverse osmosis, and electro-deionization (UF-RO-EDI) for producing demineralized water from a tertiary petrochemical effluent. The cost of the production of demineralized water (90 m^3^/h) was 0.64 USD/m^3^, and the water quality resulted in water boiler reuse.

The collected information shows that the cost of water recovery is affordable for the industries that have possibilities of water reuse, and thus it is justifiable when large quantities of water are required for use in production or when the industries are experiencing water shortage problems. In addition, the costs of recovering water from industrial effluent treatment also are environmentally valid.

In the present research, the treatment of a food industrial effluent for water recovery was evaluated to determine the technical and economic feasibility of the implementation of the project at the industrial level. The evaluation consists of an analysis of the coupled processes of membranes to enhance the efficiency of the treatment and the costs of water recovery.

## 2. Materials and Methods

### 2.1. Materials and Reagents

The reagents were used for the physicochemical determination of the quality characteristics of the industrial effluent, treated effluent, and recovered water. All reagents were analytical grade, including H_2_SO_4_ (Fermont, 97.7%, Monterrey, Nuevo León, Mexico), Hg_2_SO_4_ (Fermont, 99.9%, Monterrey, Nuevo Leon, Mexico), Ag_2_SO_4_ (Merk, 100%, Naucalpan State of Mexico, Naucalpan, Mexico), K_2_CrO_4_ (Merk, 99.3%, Naucalpan State of Mexico, Mexico), AgNO_3_ (J.T. Baker, 99.0%, Xalostoc, Mexico City, Mexico) (Meyer, 99.3%, Tlahuac, Mexico City, Mexico), and K_2_Cr_2_O_7_, and were purchased from J. T. Baker. A stock of digesting solution, acid solution, and a solution of AgNO_3_ were prepared and stored in the dark at room temperature.

### 2.2. Evaluation of the Technical Feasibility of the Treatment Process of Industrial Effluent for Water Recovery

The treatment process for water recovery includes coupled membrane processes, evaluating their technical feasibility for implementation at industrial level, using the following criteria. (1) Assessment of the physicochemical characteristics of the industrial effluent. Data on the characteristics of the effluent were collected to determine the quality of the effluent and establish the treatment process to purify the water. (2) Design and application of the treatment process of the industrial effluent treatment for water recovery. In this step, the selected treatment was evaluated at laboratory level to design the process and collect data on its application. (3) Evaluation of the effectiveness of the treatment. The efficiency of the treatment process was evaluated on the impact on the RO membrane, measuring the water quality characteristics and membrane efficiency. (4) Design of the plant for water recovery at industrial dimensions. The plant was designed at an industrial level, scaling up the data obtained on a laboratory scale to an industrial scale to integrate the plant equipment by reviewing the technology existing on the market to include that which meets industrial needs.

#### 2.2.1. Physicochemical Characteristics Assessment of the Industrial Effluent

The effluent under study comes from the food additives industry, which produces synthetic food colourants.

**Figure 1:**
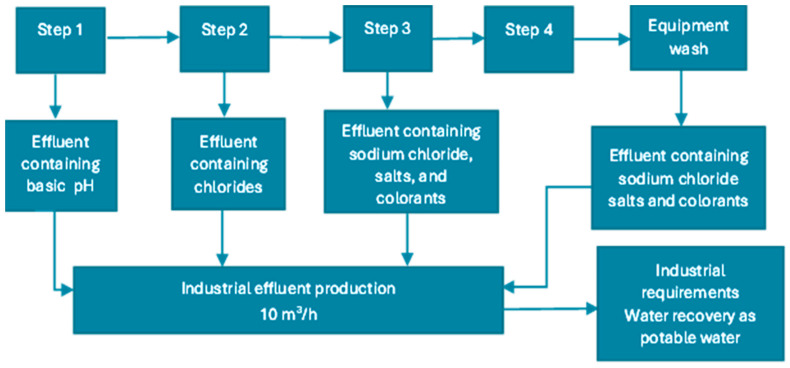
Origin of industrial effluent for treatment and water recovery.

Figure 1 exposes the origin of the effluent, which is produced by four steps of colourant manufacture, generating in average 10 m^3^/h of effluents.

The effluent consists of a mixture of wastewater containing colourants, chlorides, and sodium salts. Water from the washing equipment also contributes to the effluent mixture.

The industry requires effluent treatment for water recovery with potable water characteristics for reuse.

The measuring of industrial effluent composition was carried out on recollected sampling of the effluent mixture, including four samples for 1 month. The four samples represent the data variation from the previous screening of samples.

The samples were stored in 1 L containers and kept in refrigeration at 4 °C. The physicochemical characterization of the effluents included the following parameters: pH, electrical conductivity, EC (mS/cm), Total solids, TS (g/L), Total Dissolved Solids, TDS (g/L), Chemical Oxygen Demand, and COD (g/L). The procedures for effluent characterization are described in Standard Methods for the Examination of Water and Wastewater [16]. The equipment used to measure the physicochemical parameters were a HANNA benchtop multiparameter meter brand HI5522-01 (Woonsocket, RI, USA), a Perkin Elmer UV/Vis Spectrophotometer, Lambda 35 (Waltham, MA, USA), a potentiometer with a pH HI1131B electrode BNC (Woonsocket, RI, USA), and a Riossa drying oven model H-33 (Monterrey, Nuevo Leon, Mexico).

Due to the observable colour of the effluent, “True colour” also was measured using the indicated methodology in the Mexican Norm (NMX-AA-017-SCFI-2021) [17].

According to NMX-AA-017-SCFI-2021, true colour in water is described as the observed colour that remains in dissolved form after passing through a 0.45 µm membrane filter. Therefore, True colour was determined on a water sample previously filtered by a 0.45 µm membrane filter, measuring the spectral absorption coefficient α(λ), which is expressed in m^−1^ and is calculated using Equation (1), where A is the colour absorbance of the sample at the wavelengths (λ) of 436, 525, and 620 nm, d is the light path of the cell, in millimetres, and f is a factor used to obtain the spectral coefficient, in m^−1^ (f = 1000). The A was measured in a spectrophotometer PerkinElmer, Lambda 35, EUA.

(1) $$\alpha (\lambda )=(\frac{A}{d}f)$$

Table 1 shows the value ranges of the physicochemical parameters of the industrial effluents, linked to the four samples measured.

According to the Mexican Norm NOM-001-SEMARNAT-2021 [18], the effluent exhibited high values of True Color, which are outside the range of maximum limits in clean water for reuse (7 m^−1^ at 436 nm; 5 m^−1^ at 525 nm; 3 m^−1^ at 620 nm), indicating a highly polluted effluent by colour.

Consistent with the origin, the effluent colour exposes a mixture of colourants with intense red colouration, absorbing light in the spectra of visible regions 436, 525, and 620 nm. The colourant combinations resulted in food azo dyes, comprising Tartrazine (1-(4-sulfonatophenyl)-4-(4-sulfonatophenylazo)-5-pyrazolone-3-carboxylate), Allura red (Disodium6-hydroxy-5-[(2-methoxy-5-methyl-sulfonatophenyl)diazenyl] naphthalene -2-sulfonate) and Brillant blue (disodium;2-[[4-[ethyl-[(3-sulfonatophenyl)methyl]amino]phenyl]-[4-[ethyl-[(3-sulfonatophenyl) methyl] azaniumyl Dene] cyclohexa-2,5-dien-1-ylidene] methyl] benzenesulfonate), providing yellow, red, and blue colouration.

In turn, COD in the effluent was linked to organic mixtures of the colourants and other residual raw materials employed in the colourant manufacturing.

The pH of the effluent was basic, due to the water from step 1 (Figure 1) from colourant manufacture. The value of EC indicated a high containment of the inorganic salts, predominating sodium chloride salts (Volhard test). Consequently, TS and TDS also were associated with dissolved salts of sodium chloride. The salts are from steps 2–4 of food colourant manufacture (Figure 1).

According to the last information, the industrial effluent was declared to be dyed water, containing azo food dyes, and saline characteristics with predominant sodium chloride salts.

#### 2.2.2. Design and Application of the Process of Effluent Treatment for Water Recovery

Concerning the effluent content, the design of the treatment process for water recovery consisted of colour removal and desalination, using three alternatives of treatment at the laboratory scale. (1) Single reverse osmosis (SRO) applied the membrane process for the joint removal of colourants and salts. (2) By coupled processes, using the previous step of the chemical oxidation of colourants by single ozone (SO) and RO for desalination, resulted in SO-RO. (3) A coupled process comprising ozonation catalyzed by activated carbon (ACO) [19] and RO for salt removal is indicated as ACO-RO.

**Figure 2:**
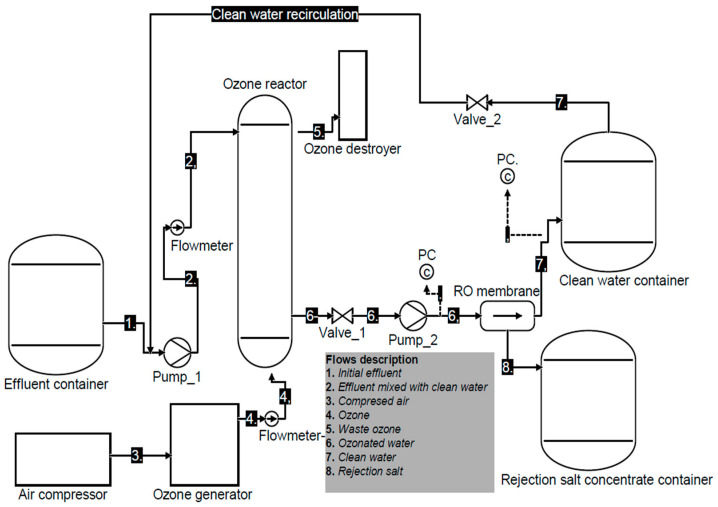
Diagram of the process for water recovery by effluent treatment using ACO-RO.

Figure 2 shows the diagram of the alternative treatment ACO-RO for water recovery. The diagram of SO-RO is like ACO-RO; however, SO-RO does not contain activated carbon (AC) in the column of ozone, while RO does not contain the step of ozonation.

The ACO-RO process included separation equipment’s and flows. Line 1 supplies the initial effluent; line 2 provide diluted effluent, due to mixture of initial effluent with clean water recirculation from RO permeate; line 3 correspond to air from compressor; line 4 supplies ozone reactor; line 5 transports waste ozone; line 6 supplies RO system with treated effluent by ozone; line 7 transport permeate stream from RO, consisting in clean water; line 8 transports salts rejection from RO.

The ozonation processes operated under similar conditions as the flux ozone with the difference being the use of AC in ACO-RO, whereas RO was similar in all tests.

Table 2 exhibits the selected operating conditions used in the effluent treatment for water recovery by SRO, SO-RO, and ACO-RO.

The ozone processes concern the ozone generator from the air, the pump of effluent feed, and reactor columns of ozonation. Ozone was supplied in continuous flow in a glass column to carry out the degradation of the dyes in the batch process until the maximum colour was degraded and removed.

The RO process involves effluent desalination, containing a commercial membrane of polyamide, operating in continuous form and in tangential flow. The RO membrane is fed by a peristaltic pump in a high-pressure range.

Using RO, we obtained two streams: the permeate, and rejection. The permeate stream is clean water produced by the processes. The process proposal includes the recirculation of 30% of clean water from the membrane permeate in the fed of SO, ACO, or SRO to reduce the concentration of salts and colouration in the effluent, maintaining the membrane operation in a continuous flow and avoiding fast membrane fouling.

The rejection stream is a concentrated effluent containing high salinity, which is disposed for reuse. Both rejection and permeate are recollected in storage recipients.

The experimental tests of treatment processes were run by treating 1 L of effluent.

The quality characteristics of recovered water were measured under similar parameters of industrial effluent using Standard Methods [16]. The efficiency of treatments was determined by membrane flux and membrane fouling.

#### 2.2.3. Plant Design of the Treatment Process of the Effluent for Water Recovery at Industrial Dimensions

Plant design at industrial dimensions was carried out by scaling up to a laboratory system of treatment using a similarity method and maintaining the operating conditions and the quality characteristics of the recovered water at the industrial level [5,20].

Equation (2) describes a similitude ratio between two different sizes. Condition (1) involves the ratios of information obtained at laboratory level, for example, fed flow of effluent (FF in m^3^/h) and the area or dimensions of the process equipment at laboratory size (A in m^2^). Condition (2) exposes the fed effluent (FF in m^3^/h) at industrial level and area (A in m^2^) or dimension of the equipment at industrial size for each step of the treatment.

(2) $$({\frac{FF}{A})}_{1}=({\frac{FF}{A})}_{2}$$

For the scaling up of ozone equipment, four important points were considered: (i) required ozone demand (g/m^3^) at laboratory test; (ii) the flow of effluent to be treated at industrial level (Scaling up flux = 10 m^3^/h); (iii) ozone compensation (25%), due to the decomposition half-life of ozone in 15 min as 0.25 h; and (iv) the equipment acquisition should include 20–50% more of the ozone dose required at industrial level. The calculi of the ozone dose were carried out by Equation (3) [21].

(3) $$\mathrm{Scaling}\;\mathrm{up}\;\mathrm{of}\;\mathrm{ozone}\;\mathrm{dose}\;(g/h)=\frac{\mathrm{ozone}\;\mathrm{dose}\;\mathrm{at}\;\mathrm{lab}\;\mathrm{scale}}{0.25\;h\;(0.5)\;}\left(\mathrm{Scaling}\;\mathrm{up}\;\mathrm{flux}\right)$$

Once the conditions of the scaled equipment were in place, it was necessary to verify if the necessary technology is commercially available. Likewise, know the legislation that governs the recovery of water from polluted effluents and the waste that may be generated by the implementation of the project. In turn, electrical energy consumption was obtained from the energy requirements specified in the technical sheets of the equipment and the costs of the energy per kWh [22], involving 16 h of operation time.

### 2.3. Economic Analysis of the Treatment Process of the Effluent for Water Recovery

The economic evaluation of the effluent treatment process involved a cost analysis of the inversion of the process for water recovery, the cost of water production/m^3^, the cost of water production/year, and the payback period to recover the cost of the inversion [5].

The financial indicators to determine economic feasibility were calculated according to Equations (4)–(8).

Cash flow:

(4) CF=I−C

Minimum acceptable rate of return:

(5) MARR=riskpremium+f+(riskpremium)(f)

Net present value:

(6) $$NPV=\sum_{t=1}^{t}\frac{CF}{{\left(1+i\right)}^{t}}-C_{0}$$

Internal rate of return:

(7) IRR=NVP=0

Payback period:

(8) $$PP=\frac{C_{0}}{\mathrm{Avarage}\;\mathrm{annual}\;\mathrm{of}\;\mathrm{cash}\;\mathrm{flow}}$$

Cash flow (CF) involves production, management, financial, and investments costs, and working capital. Minimum acceptable rate of return (MARR) represents the annual rate of profit to carry out the implementation of the project. Net present value (NPV) is the monetary value that results from subtracting the sum of the flows discounted in the present from the initial investment (considering NPV > 0 if the project is profitable; if NPV < 0, the project is not profitable, and if NPV = 0, the project can be chosen to be carried out or not). Internal rate of return (IRR) is the value of the discount rate that makes the NPV equal to zero. Payback period (PP) is the amount of time in which the investment made is recovered.

## 3. Results and Discussion

### 3.1. Technical Evaluation of the Treatment Process of Industrial Effluent Treatment

#### 3.1.1. Quality Characteristics of Recovered Water from Industrial Effluent Treatment

Table 3 exhibits the quality characteristics of recovered water by SO, SO-RO, ACO, ACO-RO, and SRO.

Regarding the pH of the treated effluent, SO and ACO showed changes in this parameter. The original pH of the effluent decreased in the SO process, while the pH value had a slight increase in the ACO treatment. The decreasing pH in SO was associated with the formation of organic and inorganic acids during the oxidation of organic matter; in contrast, the pH increase in ACO was linked to an excess of •OH [23].

In general, the colour was removed by ozonation. SO and ACO processes reduced the colouration in 95–98%, fulfilling the objective of this step of treatment.

The red and blue colours were primarily degraded by SO and ACO. After, both treatments displayed a residual yellow colouration, indicating the superior stability of yellow colouration during the treatment by SO.

The yellow permanency in the treated effluent by SO and ACO was attributed to tartrazine content, azo groups, and sulfonic acid groups that are difficult to degrade by ozone [24].

Although both processes, SO and ACO, were effective for colour removal, they observed differences in the time of treatment. The change in colouration during the application of SO was observed in 8 h, while ACO showed 1 h of batch treatment. The short time of colour elimination by ACO was linked to the catalyst and adsorption by AC [19], resulting in an effective process of oxidation for the colour degradation of the industrial effluent.

The short times of colour removal by SO and ACO was favoured by basic pH because ozone produces •OH radicals, predominating a colourant attack of •OH radicals than molecular ozone, as in O_3_. The production mechanism of •OH production by alkaline pH is according to reactions (1)–(4) [9,24].
$O_{3}+{OH}^{-}\to {HO}_{2}^{-}+O_{2}$(1)${•O}_{3}^{-}\to O_{2}+{•O}^{-}$(2)$O_{3}+{HO}_{2}^{-}\to {•HO}_{2}+{•O}_{3}^{-}$(3)${•O}^{-}+H_{2}O\to •OH+{OH}^{-}$(4)

In turn, Table 4 exposes the mechanisms of colourant removal by ozone, using SO and ACO processes.

The principal mechanism of colourant removal in SO is given by the colourants’ interaction with •OH (reaction (5); however, molecular ozone also could act in the colourant degradation, as is indicated in reaction (6).

Due to the contact between the catalysts AC, •OH, and O_3_, the mechanisms of colourant removal in ACO are based on three oxidation reactions which are mostly carried out in AC, because the colourants and O_3_ are adsorbed in this matrix. Reaction (7) indicates the adsorption of colourants on AC surface. After, reaction (8) exposes the adsorbed colourant attack by •OH, forming oxidized compounds by catalytic action. Reaction (9) shows the oxidation of adsorbed colourants in AC by molecular ozone attack, also producing oxidized compounds by catalysis of AC. Reaction (10) is presented when molecular ozone is attached on AC to produce •OH species by catalysis. In addition, the colourants are degraded likewise in reaction (5) [25,26].

In order, the total colour removal was achieved by coupled processes SO-RO and ACO-RO and single SRO, showing no detected colour (ND) in clean water because the RO membrane has a capacity for colour rejection. The mechanism of colourant removal in the RO membrane is due to diffusion. Consequently, colourant removal in SRO was performed by the diffusion procedure.

Consistent with colour removal, ACO and SO treatments also were capable of COD elimination in the treated effluent. The times of the COD treatment were similar to the colouration. The COD degradation by ACO was 1 h, while the degradation of COD by SO was 8 h. In turn, RO in SO and ACO completed COD removal because the COD measure was not detected (ND), while SRO removed 90% of COD. Therefore, it was observed that in the previous step of ozonation, SO and ACO enhanced the COD removal and the RO membrane efficiency.

In order, the desalination step by RO complemented the effluent treatment of the coupled processes SO-RO and ACO-RO; however, by ACO, salt content was also reduced because its was also adsorbed on AC. Consequently, SO and ACO alleviated the RO process, generating clean water with the highest quality and reuse possibilities. Clean water covered the range requirements of potable water quality of pH (6–8) and TDS (1 g/L) [27]; however, single RO (used as control process) showed 1 g/L of TDS, exceeding international water quality regulations of salt content.

Considering the above information, the treatment processes SO-RO and ACO-RO, including SRO, were suitable for effluent treatment and water recovery. However, coupled processes were most efficient, because the previous steps of SO or ACO enhanced the RO membrane behaviour. Despite this, due to the time of treatment, ACO-RO was considered the most satisfactory process because the colour treatment was 30 min and ACO reduced 7% of salts due to the adsorption capacity of AC, enhancing the efficiency of RO process.

Some research on effluent treatment by the coupled process of ozonation and RO were found in the literature, showing similar results to those found in the present investigation. Mohan and Oke [9] treated a textile effluent using ozone oxidation as a previous treatment of RO, conforming a coupled process, SO-RO. As a result, they achieved 94.6% of colour removal and 67.4% of COD. Cardoso et al. [28] used catalyzed ozonation by carbon-doped copper (II) to degrade four dyes of textile effluent (Methyl Orange, Orange II sodium salt, Reactive black 5, and Remazol Brilliant Blue). The efficiency of colour removal was 90% in dye degradation in 6 min for azo dyes and 30 min for anthraquinone dyes. Vega-Álvarez et al. [19] investigated the application of catalyzed ozone by AC (O_3_-AC) to degrade colourants from additives in food wastewater, reporting 99% colour and COD removal in 6 min of treatment. Cui et al. [29] researched and developed the application of an ozone catalyst RO-MCO (Cu/Fe/Ce-CAC) to degrade the pollutants of high chromaticity and complex composition. The membrane efficiency for COD removal was 54.51%, reaching total degradation < 90 min of treatment.

Nzaba et al. [30] conducted a comparative study between single ozonation and catalytic ozonation with BiOI (bismuth oxoiodides) and N, Pd-TiO_2_ (co-doped semiconductor) as a catalyst and visible light to degrade methylene blue (MB), reporting a degradation of 92% for single ozonation (SO) and 97.2% with Biooil-ozone and catalyzed ozone with light, resulting in 100% of colour elimination in 8 min.

#### 3.1.2. Efficiency of the Treatment Process of the Effluent for Water Recovery

**Figure 3:**
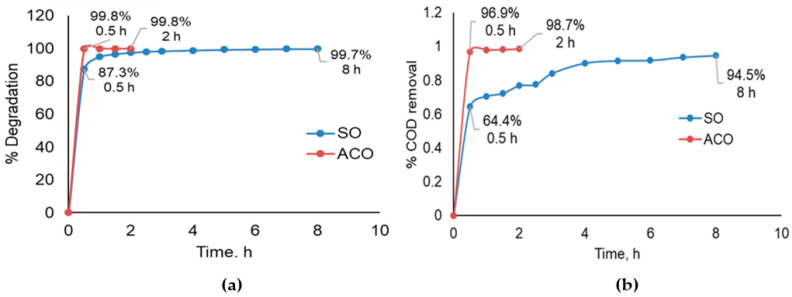
(**a**) Kinetic of % of colour degradation and (**b**) kinetic of % of COD removal of food effluent treatment with SO and ACO.

1. Efficiency of the oxidation process for colour degradation. Figure 3 confirms the last data obtained using the kinetic of colour and COD removal.

ACO reduced the colouration and COD efficiently (98%), degrading the colour and other organic compounds in a short time (1 h), which was attributed to the catalytic action of AC on the ozone and the adsorption capacity of AC [19]. Oxidation by SO was also efficient in colour degradation; however, 94% of oxidation was achieved in 8 h of treatment.

**Figure 4:**
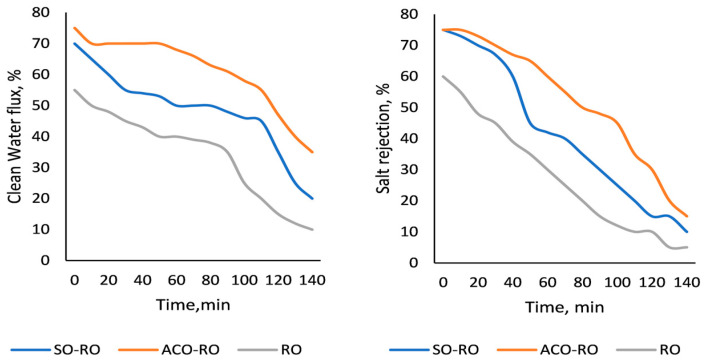
RO membrane efficiency from the SO-RO, ACO-RO, and SRO treatment processes by clean water flux (%) and salt rejection (%).

2. Efficiency of the desalination process and water recovery. Figure 4 exhibits the RO membrane behaviour during effluent desalination of the effluent by SRO and the treated effluent by the SO-RO and ACO-RO coupled processes, showing efficiency ranges in salt removal of 55–75%.

The most efficient process for desalination was ACO-RO, producing 6.5–7.5 m^3^/h of clean water, equivalent to 70–75% of efficiency in water recovery by permeate flux and salt rejection, while SO-RO produced 6.0–6.5 m^3^/h of clean water with 60–65% of efficiency and SRO exposed 55% of efficiency with 5–5.5 m^3^/h.

In addition, Figure 4 shows that the behaviour of membrane fouling was different in each treatment. SRO exposed the lowest flux permeate in 90 min, achieving the maximum fouling, whereas SO-RO and ACO-RO were exposed in 140 min, indicating that the most efficient process for water recovery from industrial effluent treatment was ACO-RO.

**Figure 5:**
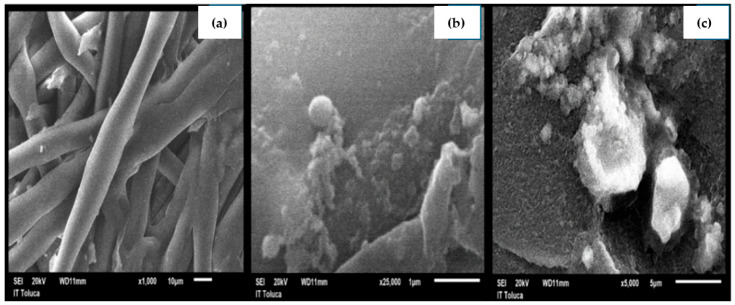
SEM images of polyamide membrane by treatment, (**a**) SO-RO, (**b**) ACO-RO, and (**c**) SRO.

Complementing the last report, Figure 5a–c exposes SEM images of the polyamide membrane, showing the status of membrane fouling after the effluent treatment by ACO-RO, SO-RO, and SRO, where it is observable that the membrane from SRO contains the highest salt accumulation than ACO-RO and SO-RO, indicating that the most exposed to fouling was the SRO process, because the previous treatment, SO and ACO, reduced the fouling by salts and colourants.

Several studies reported in the literature have focused on water recovery from saline effluents using RO and integrated systems, especially in seawater, and a few others in saline effluents from industry reporting efficiencies greater than 80% in water recovery.

Partal et al. [31] investigated the treatment of textile wastewater by coupled processes SO-NF-RO-IEX, cero residues, and 80% of clean water.

Loung et al. [32] evaluated two processes for salt removal: one coupled process at low pressure (LPRO) with membrane capacitive deionization (MCDI), and another with single RO to desalinate brackish water. By RO, the water is used as drinking water, with an efficiency greater than 99.3% of salt rejection and 43.8% of recovered water. The LPRO permeate water has a salt rejection efficiency greater than 91.6% of permeate and a salt rejection of 37.6%. The LPRO permeate water was sent to the MCDI, which had a salt rejection efficiency of 65% and recovered water of 80.6%.

In turn, Dévora-Isiordia et al. [33] determined the membrane fouling concentration factor compared to a simulator for the treatment of synthetic brackish water (13,335 mg/L of salt) and seawater (35,522 mg/L of salts) with RO. The salt rejection efficiency resulted in 99.46% at 6.07 MPa, and for seawater, the salt rejection efficiency was 99.59% at 6.06 MPa. Álvarez-Sanchez et al. [34] tested a membrane composite (TFC) to desalinate salt water and increase permeate flow through a layer of polyamide and ZnO nanoparticles (NP). The salt rejection efficiency was 97.13–97.77%. Vazquez et al. [35] synthesized a responsive membrane of polysulfone and smart polymers (PSF-SRPs) to desalinate water from the food industry effluent for water recovery and recycling, reporting the efficiency of water flux and salt rejection to be 20–30%.

#### 3.1.3. Scaling Up and Plant Design of Treatment Process of Effluent for Water Recovery at Industrial Dimensions

**Figure 6:**
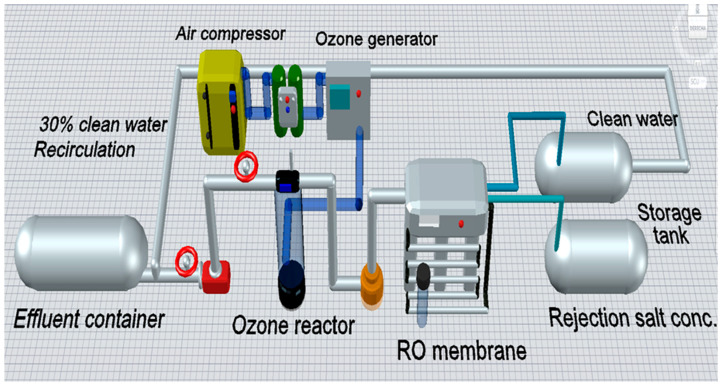
Plant design (3D) of the treatment of the industrial effluent for water recovery by the coupled processes, ACO-RO. Top: frontal view. Bottom: view from above.

Depending on the applied processes for effluent treatment, Table 5 exposes the scale-up of equipment to laboratory size to the industrial level of the process. In turn, Figure 6 shows the plant’s design (3D) of the industrial effluent by ACO-RO, including capacity measures of equipment for the degradation of colourants by ozonation and desalination of the effluent by RO.

Data of laboratory sizes are represented by feed flow FF = 0.001 m^3^/h, whereas the industrial effluent requires 10 m^3^/h of feed flow.

According to Equation (3), and considering data from the laboratory test (6.6 g/m^3^ of ozone dose and 0.25 h of half time of the ozone), ozone demand at the industrial level resulted in 265 g/h. However, the equipment acquisition included 50% of the total ozone required, exposing <530 g/m^3^. Commercially, there is equipment that produces 560 g/h of ozone.

Ozonation at industrial scale comprises an ozone generator and a column of reactor oxidation, their sizes match with similitude scaling.

Table 5 and plant design involve a system of RO at industrial level, which also was determined by similitude scaling. The RO system includes seven polymeric membranes of RO (for desalination (LGSW 440 R G2; Chem Ltd. Company, Seoul, Republic of Korea) of composite polyamide, operating in tangential flow. Table 5 shows the principal specifications of RO membranes. Data on the industrial level correspond to the adjusted information on scaling up and the available commercial equipment.

Storage tanks incorporate industrial effluent space, recovered water from membrane permeation, and the rejection from RO.

Pumps, valves, and measurement instruments were also integrated into the plant design by similitude scaling.

The size of the area of the plant to cover the industrial conditions for both processes resulted in approximately 130 m^2^. Consistent with the size of the plant and the objective of implementation, it requires three workers to operate.

Table 6 exhibits the required equipment available on the market for integrating the treatment plant at industrial dimensions and costs.

### 3.2. Economic Analysis of the Process for Water Recovery from Industrial Effluent Treatment

**Figure 7:**
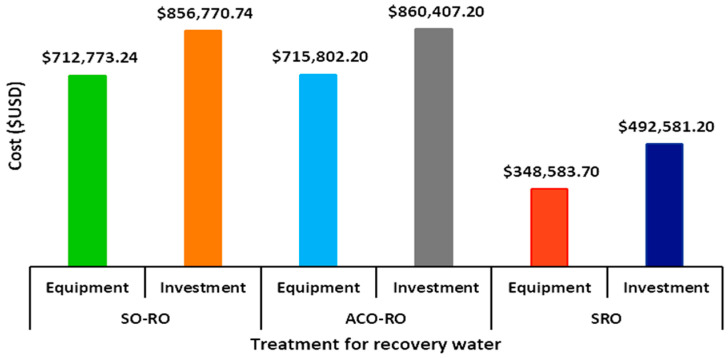
Costs of the equipment and total investment for each treatment to water recovery.

Figure 7 show the cost of the equipment and investment cost for each studied treatment.

Total investment for the implementation of alternatives to recover water from 10 m^3^/h of effluent, including construction, equipment, installation, unforeseen, direct services, raw material, working capital, and others, was USD 856,770.74 for the SO-RO system, USD 860,407.2 for ACO-RO, and USD 492,581.20 for SRO.

SRO resulted in a more economic treatment because it does not comprise the step of ozonation; however, other limitations of this process are exposed below.

Table 7 summarizes the cost of investment, costs for waste disposal, and operating costs, including the cost of electrical energy consumption (kWh) and consumables.

Data on operating costs indicate that SO-RO has the highest cost because the treatment for colour removal includes 8 h of operation, whereas colour removal by ACO-RO is 30 min. In turn, SRO has the lowest cost due to the absence of an ozonation step.

The cost of waste disposal refers to sludge disposal, exhausted AC, membranes, cleaning and maintenance, and other factors. In this case, SRO resulted in the highest cost because the treatment generates waste consistent in salt with colour, whereas SO-RO and ACO-RO generate concentrated effluent saline that is utilizable in the industry.

The minimum acceptable rate of return (MARR) was indicated as an annual rate of profit of 4.6% [36] and a risk premium of 12.5%, obtaining 17.7% for both treatments, SO-RO and ACO-RO, whereas SRO resulted in the highest values.

Including 7 years of capitalizable periods, investment (C_0_), and Cash flow (CF) of USD 856,770.74 and USD 251,395.0 for SO and USD 860,407.20 and USD 251,354.30 for ACO, respectively, the NPV calculated cost was USD 109,652.59 for SO and USD 105,859.67, for ACO. For both alternatives, the projects are economically feasible, with a payback period of 3.4 years for both processes. Furthermore, the internal rate of return (IRR) was calculated at 22.1% and 21.9%, respectively.

The cost to treat 1 m^3^ of effluent per year resulted in USD 1.4 for SO-RO and ACO-RO; however, SRO showed the highest value due to the cost of waste disposal.

According to the economic analysis, the suggested alternatives, SO-RO, ACO-RO, and SRO, were economically feasible. However, due to the time of treatment, ACO-RO resulted as the most attractive alternative because ACO degrades the colour in 30 min, matching with the information from Vega-Álvarez et al. [19], Shokouhi et al. [37], and Bilinska et al. [38].

Currently, there is effluent treatment information with coincident data from this investigation. Zagklis and Bampos [39] reported the costs of the processes of oxidation, including ozone and Fenton as tertiary treatments of the effluents from WWTP. Ozone oxidation resulted in 0.07 USD/m^3^ and Fenton reported a cost of 1 USD/m^3^, showing that ozonation is the most economical oxidation process.

Regarding comparable treatment costs for the coupled processes of membranes, Sewilam and Al Bazedi [40] developed two integrated systems with Fenton oxidation, FO-RO, to treat prepared wastewater with different hard concentrations. The costs resulted in a range of 1.8–1.9 USD/m^3^, indicating the high benefits of the wastewater treatment.

Another process cost based on membrane treatment was reported by Pérez et al. [41]. They tested a coupled system formed by UF-RO to treat wastewater from a secondary effluent from WWTP. The cost was 12,5 USD/m^3^, recovering 10.8 m^3^/h of clean water from 20 m^3^/h of effluent. In turn, Wu et al. [42] proposed an integrated system, including forward osmosis and membrane distillation (FO-MD) processes to treat textile wastewater. FO membranes were doped with graphene oxide (GO) and compared with a commercial membrane. The estimated water production cost of water recovery using a doped membrane was 6.48 USD/m^3^.

## 4. Conclusions

The coupled membrane processes SO-RO and ACO-RO for industrial effluent treatment were technically and economically feasible for its implementation at industrial level. The following arguments were found during the project development.

(1) The principal constituents of the effluent were colourants and sodium chloride salts; therefore, the effluent depuration for water recovery comprised colouration treatment and salt removal. (2) Colouration treatment was evaluated by the degradation in colour using single ozonation (SO) or catalyzed (ACO) ozonation by activated carbon (AC), while salt removal was suggested by reverse osmosis membranes RO, conforming coupled processes SO-RO and ACO-RO for effluent treatment, and, as a process control, single RO. (3) The ozonation processes SO and ACO were efficient for colour degradation; however, ACO degraded the colour in 30 min, whereas SO was in 8 h. (4) In comparison with single RO (SRO), both ozonation processes increased the efficiency of the membrane; however, ACO-RO was the most efficient process, increasing the membrane flux and water recovery with fouling decrease. (5) The scaling up of SO-RO and ACO-RO processes resulted in a plant design for 10 m^3^/h of effluent treatment. Using ACO-RO, the water recovery with clean production was 7.7 m^3^/h. The plant size was 130 m^2^. (6) The equipment of the plant is commercially available; therefore, the integration and implementation of the treatment plant at industrial level is viable. (7) The cost of treatment using ACO-RO resulted in 1.4 USD/m^3^, and the time of cost recovery by investment was 3.4 years, which is comparable with previous studies. Technical and economic analyses showed the viability of the project of industrial effluent treatment.

## Figures and Tables

**Table 1:** Physicochemical characteristics of the industrial effluent.

Parameter	pH	EC (mS/cm)	TS (g/L)	TDS (g/L)	COD (g/L)	True Colour (m^−1^). Red Colouration Predominancy		
						436 nm	525 nm	620 nm
Range values	8–9	37–40	30–35	30–35	4–5	660 ± 5	552 ± 3	210 ± 8

**Table 2:** Ranges of operating conditions of laboratory-scale processes SO-RO and ACO-RO for water recovery from effluent.

Oxidation System						
Ozone process	Column volume (m^3^)	Fed flow effluent (m^3^/h)	Operating temperature range (°C)	Effluent pH	Ozone flow (g/m^3^)	AC volume (m^3^)
SO	0.0012	0.001	18–21	8–9	6.6	0
ACO	0.0012	0.001	18–21	8–9	6.6	0.0003
RO System						
RO process	Material	Lineal Flow velocity (m^3^/m^2^h)	Operating temperature and pressure range	pH	Feed flow effluent (m^3^/h)	Percentage of Permeate
Membrane process	Composite polyamide membrane 0.030 m^2^	<0.04	21–24 °C 9–10 bar	8–9	<0.001	<75

**Table 3:** Range values of physicochemical characteristics of treated effluent and recovered water by SRO and coupled process, SO-RO and ACO-RO.

Parameters		Fed Industrial Effluent	Treated Industrial Effluent				
			SO	ACO	SO-RO	ACO-RO	SRO
pH		7.5–8.0	7.0–7.5	8.5–9.0	7.0–8.0	7.0–8.0	8.0–9.0
EC (mS/cm)		26–30	25–30	18–20	0.5–1.0	0.2–0.5	3–5
TS (g/L)		20–25	20–25	18–24	1.0–1.2	0.6–1.0	1.0–1.5
TDS (g/L)		20–25	20–25	18–20	0.8–1.0	0.6–0.8	1.0–1.5
COD (g/L)		3–4	0.5–1.0	0.1–0.2	ND	ND	1.0–2.0
True colour m^−1^	436 nm	560–563	15–20	5–10	ND	ND	ND
	525 nm	452–455	3–4	0–1	ND	ND	ND
	620 nm	110–111	2–3	1–2	ND	ND	ND

ND: Not detected.

**Table 4:** Mechanisms of colourant removal by SO-RO, ACO-RO, and SRO.

SO	Colourants + •OH → Oxidized products	(5)
	Colourants + O_3_ → Oxidazed products	(6)
ACO	AC+colourants→AC−colourants	(7)
	AC−colourants+•OH→Oxidizedproducts	(8)
	$AC-Colourants+O_{3}\to Oxidazedproducts$	(9)
	$AC+O_{3}\to •OH$	(10)

**Table 5:** Scale up the laboratory system to the industry level for effluent treatment by ACO-RO and SO-RO for water recovery.

Process	ACO-RO		SO-RO		SRO	
Equipment and Consumables	Laboratory	Industrial	Laboratory	Industrial	Laboratory	Industrial
Feed flow of industrial effluent (m^3^/h)	0.001	10	0.001	10	0.001	10
Ozone column volume (m^3^)	0.0012	6	0.0012	6	0	0
Ozone flow (g/h)	6.6	530	6.6	530	0	0
Air flow (m^3^/h)	0.003	30	0.003	30	0	0
AC volume (m^3^)	0.0003	1.5	0	0	0	0
Total active area of RO membrane (m^2^)	0.030	300	0.030	300	0.030	300
Active area/membrane	0.030	41	0.030	41	0.030	41
Diameter size of membrane (m)	0.0015	0.2	0.0015	0.2	0.0015	0.2
Number of required membranes	1	7	1	7	1	7
Membrane pressure (bar)	9	9	9	9	9	9
Clean water flux/membrane (m^3^/h)	0.0007	1.1	0.0006	1.0	0.0005	0.9
Total Clean water stream (m^3^/h)	0.0007	7.7	0.0006	6.0	0.0005	5.0

**Table 6:** Characteristics of plant equipment of the ACO-RO and SO-RO processes at the industrial level.

Stages of Treatment	Equipment	Equipment Characteristics	Required Units SO/ACO/SRO	Required Area per Unit (m^2^)	Cost (USD) per Unit
Industrial effluent	Storage tank	300 m^3^ capacity	1	60	5525.8
Ozonation	Air compressor	Capacity 1000 L Power 40 HP Pressure range < 1 bar	1	2	75,596.7
	Oxygen generator	Purity of 93 ± 3% Pressure range > 1 bar	1	2.5	69,449.7
	Ozone generator	560 g/h Pressure range < 0.6 bar	1	2	207,118.0
	Centrifugal pump	7.5 HP, Maximum flow rate of 505 m^3^/s	2	0.5	6012.6
	Tank unit (Effluent-ozone)	Filter dimensions	1	1	9041.5
	Activated carbon filter unit	Flow rate range < 12 m^3^/h	1	1	9041.5
Desalination with RO	Module with 7 RO membranes	Flow rate range < 5 m^3^/h, Pressure range < 50 bar, Power 70 HP	1	6	338,395.7
	Permeate storage tank	Capacity 200 m^3^	1	30	2331.1
	Reject storage tank	Capacity 200 m^3^	1	30	2331.1

**Table 7:** Costs of operation and investment of the treatment plant for effluent treatment at industrial level.

Concept	SO-RO	ACO-RO	SRO
Total capital investment	USD 856,770.74	USD 860,407.20	USD 492,581.20
Operating cost/year	USD 129,131.73	USD 128,483.50	USD 174,621,324.44
Waste disposal	USD 105.0	USD 145.7	>USD 3,000,000
Cost to treat m^3^/year	USD 1.4	USD 1.4	>USD 1.4
MARR	17.7%	17.7%	>17.7%
CF	USD 251,395.0	USD 251,354.30	USD 252,500.00
NPV	USD 109,652.59	USD 105,859.67	>USD 109,652.59
Payback period	3.4 years	3.4 years	>3.4 years
IRR	22.1%	21.9%	>22.1%

## Data Availability

Data are contained within the article.

## Abbreviations

AC	Activated carbon
ACO	Ozonation catalyzed by activated carbon
C	Output costs
CAT	Catalyst
C_0_	Initial investment cost
CF	Cash flow
COD	Chemical oxygen demand (g/L)
EC	Electrical conductivity (mS/cm)
F	Inflation rate (%)
FO	Forward osmosis
GAC	Granular activated carbon
I	Input costs
IEXR	Ion exchange resins
IRR	Internal rate of return (%)
MARR	Minimum acceptable rate of return (%)
MD	Membrane distillation
MF	Microfiltration
ND	Not detected
NF	Nanofiltration
NPV	Net present value
O_3_	Ozone
RO	Reverse osmosis
SEM	Scanning electron microscopy
SO	Single ozonation
TDS	Total dissolved solids (g/L)
TS	Total Solids (g/L)
UF	Ultrafiltration
USD	United States Dollar
WWTP	Wastewater treatment plant
